# The role of digital literacy and ethical awareness in nurses’ acceptance of Artificial Intelligence: a cross-sectional questionnaire survey^✰^

**DOI:** 10.1016/j.ijnsa.2026.100577

**Published:** 2026-06-01

**Authors:** Wafaa Hassan Ali Awad, Nadia Hassan Ali Awad, Heba Mohammed Alanwer Ashour, Engy AbdlRhman Khamis, Mai Mohammed Yassen, Hadaiea Ismail Abo baker Ismail, Narges Mohammed Mohammed syam, Eshrak salama Hashem

**Affiliations:** a Medical -Surgical Nursing, Faculty of Nursing, Alexandria University, Alexandria, Egypt; b IbnSina National College for Medical Studies, Jeddah, Saudi Arabia; c Nursing program, Batterjee Medical College, Jeddah 21442, Saudi Arabia; d Nursing Administration Department, Faculty of Nursing, Alexandria University, Alexandria, Egypt; e College of Nursing– Jeddah, King Saud Bin Abdul-Aziz University for Health Sciences, Jeddah, Saudi Arabia; f King Abdullah International Medical Research Center, Ministry of National Guard Health Affairs (MNGHA), Jeddah, Saudi Arabia; g Medical -Surgical Nursing, Modern University for Technology and Information; h Department of Public Health Nursing, Faculty of Nursing, King Abdulaziz University, Jeddah, Saudi Arabia; i Nursing Department, North Private College of Nursing, Arar, Northern Border, Saudi Arabia; j Medical surgical Nursing Department, College of Nursing, Jouf University, Sakaka, Saudi Arabia

**Keywords:** Digital literacy, Artificial intelligence, Nurses, Acceptance, Artificial intelligence-ethical awareness

## Abstract

**Background:**

The integration of artificial intelligence into healthcare is rapidly transforming clinical practice. Nurses’ acceptance of artificial intelligence is influenced by their digital literacy and ethical awareness, yet empirical evidence examining these interrelationships is limited.

**Objective:**

We aimed to examine the direct and indirect effects of digital literacy on nurses’ acceptance of artificial intelligence, with ethical awareness of artificial intelligence serving as a mediating factor.

**Methods:**

A cross-sectional, correlational design was conducted among 350 nurses working in medical-surgical and critical care units at El-Kasr Al-Aini hospital in Egypt from March 2025 to December 2025. Participants completed validated instruments assessing digital literacy, artificial intelligence ethical awareness, and artificial intelligence acceptance. Data were analyzed using descriptive statistics, Pearson correlations, and structural equation modeling (SEM) with bootstrapping to assess mediation effects.

**Results:**

Participants demonstrated moderate levels of digital literacy, artificial intelligence ethical awareness, and artificial intelligence acceptance. SEM analysis revealed that digital literacy had a significant direct effect on artificial intelligence acceptance (β = 0.29, *p* < 0.001) and a strong effect on artificial intelligence ethical awareness (β = 0.46, *p* < 0.001). Artificial intelligence ethical awareness significantly mediated the relationship between digital literacy and artificial intelligence acceptance (indirect effect β = 0.13, *p* < 0.001), indicating partial mediation. The model exhibited good fit indices: chi-square/df (χ²/df) = 1.98, Comparative Fit Index= 0.95, Tucker–Lewis Index= 0.93, Root Mean Square Error of Approximation= 0.057, and Standardized Root Mean Square Residual= 0.045.

**Conclusion:**

Digital literacy enhanced participants' acceptance of artificial intelligence both directly and indirectly through ethical awareness. Ethical preparedness may, therefore, be a critical factor alongside technical competence in promoting artificial intelligence adoption among Egyptian nurses.

**Relevance to clinical practice:**

Integrating digital literacy training with ethical education in Egyptian nursing curricula and continuing professional development programs may be able to facilitate safe and effective artificial intelligence adoption, supporting high-quality patient care in technologically advanced healthcare environments.

**What is already known**

• Nurses’ use of artificial intelligence depends on their digital skills and understanding of ethics.
• Ethical awareness helps ensure artificial intelligence is used safely in clinical practice.
• Digital skills alone may not be enough to adopt artificial intelligence effectively.

Alt-text: Unlabelled box dummy alt text

**What this paper adds**

• Digital literacy may help nurses accept artificial intelligence both directly and through ethical awareness.
• Ethical awareness partly explains how digital skills may lead to artificial intelligence acceptance.
• Training in both digital skills and ethics may help nurses use artificial intelligence safely and effectively.

Alt-text: Unlabelled box dummy alt text

## 1 Introduction

The rapid integration of artificial intelligence into clinical workflows is reshaping nursing practice across multiple specialties, including critical care, pediatrics, and oncology, where complex decision-making and patient monitoring demand timely, accurate, and ethical interventions (Lambert et al., 2023). Nurses in these settings must possess not only technical skills but also ethical sensitivity and positive attitudes to ensure the safe and effective use of artificial intelligence-enabled digital tools (Alruwaili et al., 2024). Researchers have indicated that digital literacy is a prerequisite for interacting with these tools, encompassing the technical, cognitive, and communicative competencies required to interpret artificial intelligence-generated outputs, evaluate information credibility, and engage in digital communication within clinical workflows (Alruwaili et al., 2024). Additionally, artificial intelligence ethical awareness influences how nurses judge the appropriateness, fairness, and safety of artificial intelligence-enabled tools, shaping their acceptance and adoption (Alowais et al., 2023). These processes are further mediated by perceived usefulness, trust, and organizational factors that create enabling conditions for technological integration (Alruwaili et al., 2024). By explicitly linking artificial intelligence to the broader concept of digital tools. We aimed to clarify how nurses’ competencies and ethical preparedness jointly contributed to the successful adoption of artificial intelligence-enabled technologies in specialty nursing practice.

### 1.1 Theoretical framework

We drew on the Technology Acceptance Model and Rest’s Ethical Decision-Making Theory to examine the relationships among digital literacy, artificial intelligence ethical awareness, and artificial intelligence acceptance among nurses (Davis, 1989; Rest, 1986). According to the Technology Acceptance Model, individuals’ perceptions of usefulness and ease of use determine their intention to adopt new technology (Eldesoky et al., 2025). In nursing, digital literacy enhances these perceptions: nurses with higher digital competence are better equipped to navigate artificial intelligence tools, understand their functionalities, and apply them effectively in clinical practice, which increases their perceived usefulness and ease of use, thereby promoting adoption (Lambert et al., 2023; Eldesoky et al., 2025).

However, the Technology Acceptance Model does not account for ethical considerations, which are critical when artificial intelligence systems influence patient care (Ball and Michalowski, 2024). Rest's 1986 Ethical Decision-Making Theory addresses this gap by emphasizing moral awareness as the first step in ethical decision-making. In artificial intelligence-enabled nursing practice, nurses with strong ethical awareness can identify potential issues related to privacy, fairness, accountability, and transparency, ensuring that technology use aligns with professional and ethical standards (Ball and Michalowski, 2024; Kwak et al., 2022).

**Fig. 1:**
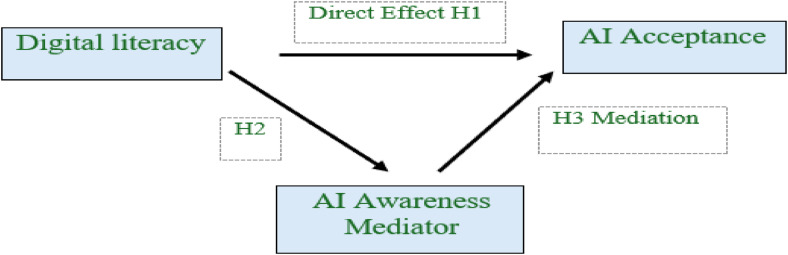
Researchers’ conceptual framework.

Based on the theoretical framework, we formulated the following study hypotheses. Digital literacy provides the technical competence required to engage effectively with artificial intelligence, while ethical awareness filters the application of this competence through a moral and professional lens. Therefore, we hypothesized that digital literacy would have a direct positive effect on nurses’ acceptance of artificial intelligence (H1). We further hypothesized that digital literacy would positively influence artificial intelligence ethical awareness among nurses (H2). In addition, we proposed that artificial intelligence ethical awareness would mediate the relationship between digital literacy and nurses’ acceptance of artificial intelligence, such that higher digital literacy leads to greater ethical awareness, which in turn increases acceptance of artificial intelligence (H3). These hypothesized relationships are illustrated in Fig. 1.

### 1.2 Digital literacy

Digital literacy is a multifaceted and evolving construct; however, there is no universally agreed-upon definition within the nursing literature. Current researchers have conceptualized it in diverse ways, encompassing the technical skills, cognitive abilities, and socio-communicative competencies required to effectively engage with digital technologies in healthcare settings (Amin et al., 2025; Mo et al., 2025). This variability reflects the rapid advancement of digital health innovations and the expanding role of nurses in technology-mediated care, particularly regarding artificial intelligence integration. In response, we have adopted a multidimensional perspective, defining digital literacy as the integration of technical proficiency, information processing abilities, and communication skills necessary for the effective and safe use of digital tools in clinical practice, including higher-order skills, such as interpreting digital outputs, evaluating information credibility, and engaging in digital communication within healthcare environments (Amin et al., 2025; Mo et al., 2025).

Within this framework, artificial intelligence is conceptualized as a type of digital tool, while artificial intelligence-enabled tools refer to applications that incorporate artificial intelligence functionalities within broader clinical software (e.g., clinical decision support modules), and artificial intelligence systems denote standalone technologies providing analytics or predictive outputs. To operationalize this construct, we used the Digital Literacy Scale by Reddy et al. (2023), a multidimensional instrument comprising six domains: media literacy, computer literacy, information literacy, visual literacy, and communication literacy. These domains collectively capture competencies required for nurses to interact with digital systems and artificial intelligence-enabled tools, supporting clinical decision-making and safe digital communication (Abou Hashish and Alnajjar, 2024).

Researchers have indicated that nurses with higher digital literacy demonstrate greater confidence using digital technologies, more positive attitudes toward innovation, and stronger intentions to adopt artificial intelligence-enabled tools and digital systems (Li et al., 2025). However, variability persists across roles and regions; for example, nurse managers often demonstrate higher familiarity with information systems, data analytics platforms, and artificial intelligence-supported workflow tools, whereas bedside nurses may primarily use digital tools for documentation and routine patient monitoring (Eldesoky et al., 2025; American Nurses Association, 2022). These disparities highlight the possible need for role-specific education and training to enhance readiness for digital transformation.

### 1.3 Ethical awareness of artificial intelligence

Ethical awareness of artificial intelligence in nursing denotes sensitivity to ethical issues that arise from artificial intelligence use, including privacy, bias, transparency, accountability, and patient autonomy, and the ability to reflect on and act according to ethical principles when artificial intelligence tools are involved (Migdadi et al., 2024). The American Nurses Association has formalized ethical guidance, signaling that artificial intelligence ethical competence is now essential to nursing practice (Suand Chao, 2022). Empirical researchers have concluded that, while many nurses recognize ethical issues associated with artificial intelligence, their depth of understanding and preparedness to act vary (Tun et al., 2025; El Arab et al., 2025). Kwak et al. (2022), in a cross-sectional study, found moderate levels of artificial intelligence ethical awareness among practicing nurses and nursing students in Korea.

Ethical awareness actively influences nurses’ appraisal of artificial intelligence acceptability (Ball and Michalowski, 2024). Technically proficient nurses may reject or mistrust artificial intelligence when it is perceived as ethically problematic, such as when transparency is lacking or patient autonomy is compromised (Asal et al., 2025). Thus, ethical awareness is not merely a background trait but a dynamic factor shaping decision-making and the intention to adopt artificial intelligence-enabled digital tools in clinical practice (Davis, 1985; Ball and Michalowski, 2024)

### 1.4 Artificial intelligence acceptance in nursing

Artificial intelligence acceptance refers to the extent to which nurses intend to use and adopt artificial intelligence technologies in clinical practice. The literature on technology acceptance models has been adapted to artificial intelligence contexts; perceived usefulness, perceived ease of use, trust, perceived risk, and facilitating organizational conditions repeatedly predict acceptance (Lambert et al., 2023; Al-Olaimat et al., 2025). Integrative reviews and empirical researchers have shown a complex picture where many nurses see artificial intelligence as an opportunity for improving care quality and efficiency, but concerns about job displacement, lack of trust, limited knowledge, and ethical uncertainty hinder acceptance (Lambert et al., 2023 & Alruwaili et al., 2024). Some researchers have suggested that trust in artificial intelligence, shaped by transparency and explainability and ethical acceptability, particularly fairness and accountability, is a central mediator between competence and actual adoption in healthcare (Lambert et al., 2023; Reddy et al., 2023). Building acceptance requires multiple factors: technical training to increase perceived ease and competence, leadership and policy support to create enabling conditions, and ethical governance to build trust (Al-Olaimat et al., 2025). Interventions that address knowledge, skills, and ethics are the most promising (Lambert et al., 2023).

Researchers have further contextualized these findings within nursing practice. Sarıkahya et al. (2025) reported that nursing faculty perceptions of artificial intelligence tools, such as ChatGPT, significantly influence curriculum integration and student readiness, highlighting the role of educational support in shaping artificial intelligence adoption. Boztepe et al. (2025) found a positive relationship between nursing students’ digital literacy and attitudes toward medical artificial intelligence, underscoring the importance of digital competence in artificial intelligence acceptance. In pediatric oncology care, Özbay et al. (2026) demonstrated that nurses’ ethical awareness, including accountability, transparency, and patient-centered decision-making, directly affects artificial intelligence utilization. These studies collectively indicate that both technical competence and ethical preparedness are essential for successful artificial intelligence adoption in nursing (Reddy et al., 2023; World Medical Association, 2013).

### 1.5 Significance

The integration of artificial intelligence into healthcare is increasingly shaping clinical workflows across a range of settings, including high-acuity environments, such as critical care, as well as general medical-surgical departments, where nurses manage complex patient needs and high workloads (Lambert et al., 2023). While artificial intelligence does not replace clinical judgment, it has the potential to augment nurses’ decision-making, enhance efficiency, and support patient monitoring and risk identification (Lambert et al., 2023). Therefore, nurses’ acceptance of artificial intelligence technologies is an important factor influencing their effective and ethical use in diverse clinical contexts (Lambert et al., 2023). The World Health Organization (2021) has emphasized that artificial intelligence for health must be designed and implemented to respect fundamental human rights, dignity, and moral standards. Furthermore, these systems should actively promote accountability, responsibility, safety, justice, equity, transparency, and inclusivity (Tekkeşin, 2019).

Although nurses are often discussed broadly in literature, we focused specifically on those working in critical care and medical-surgical units due to the clinical significance of these settings. These environments are characterized by complex patient care, high workloads, and frequent decision-making demands, where digital technologies may play a supportive role in enhancing nursing practice (Oruchkwu, 2025; Bodur et al., 2025). Most researchers have focused on students or general populations, leaving a gap in understanding how digital literacy and ethical awareness interact among practicing nurses in these contexts. Moreover, ethical awareness as a potential mediating factor between digital literacy and artificial intelligence acceptance has not been thoroughly examined. Addressing this gap is essential, as identifying and quantifying these relationships can inform education, policy, and organizational strategies to support the safe, effective, and ethically responsible integration of artificial intelligence in nursing practice.

### 1.6 Objectives of the study

We aimed to examine the direct and indirect effects of digital literacy on nurses' acceptance of artificial intelligence, with artificial intelligence ethical awareness serving as a mediating factor.

## 2 Design and methods

### 2.1 Setting and sample

A cross-sectional, correlational study was conducted across all medical-surgical and critical care inpatient units at one of the El-Kasr Al-Aini hospitals in Egypt during the period from March 2025 to December 2025 using a non-probability convenience sample of 350 nurses selected from a total of 1500 nurses with at least 6 months of experience distributed as follows: 125 nurses from medical care units, 105 from surgical care units, and 120 from critical care units. The distribution was estimated using G*Power for Windows (version 3.1.9.7), with a power of 0.95, an effect size of 0.5, and an alpha level of 0.01.

### 2.2 Data collection tools

Three validated instruments were employed to collect the required data, each measuring a distinct construct relevant to the study. These instruments were originally developed in English and were translated into Arabic for use in the present study following standard translation procedures.

#### 2.2.1 Artificial intelligence acceptance scale

The Technology Acceptance scale, originally developed by Davis (1985) and adapted by Al-Olaimat et al. (2025), has a Cronbach’s alpha coefficient of 0.92. It includes 16 items grouped into three key constructs to measure technology acceptance: perceived usefulness (6 items), perceived ease of use (6 items), and intention to use (4 items). All items are rated on a 5-point Likert scale ranging from 1 = extremely unlikely to 5 = extremely likely. The overall score ranges from 16 to 80. A higher score indicates greater acceptance of artificial intelligence. The scale demonstrated high internal consistency reliability, with a Cronbach’s α = 0.92 (Alowais et al., 2023; El Arab et al., 2025).

#### 2.2.2 Digital literacy scale

Reddy et al. (2023) created this scale to assess digital literacy. The 60 items on the scale are divided into six sub-dimensions: media literacy, computer literacy, information literacy, visual literacy, and communication literacy. These domains evaluate nurses’ abilities to access and critically evaluate digital content (media literacy), operate digital devices and systems (computer literacy), locate and manage information (information literacy), interpret visual data such as charts and interfaces (visual literacy), and communicate effectively using digital platforms (communication literacy). The 5-point Likert ranges from 1 (no understanding) to 5 (very high, expert). The overall score ranges from 60 to 300. A higher score means greater understanding of digital literacy. The scale demonstrates high internal consistency reliability with a Cronbach’s α = 0.90 (Reddy et al., 2023).

#### 2.2.3 Ethical awareness regarding artificial intelligence scale

This tool, adapted from Ko and Leem (2021), includes a 12-item scale to assess nurses’ ethical awareness regarding the intended use of artificial intelligence. The scale is organized into four dimensions: accountability, safety, transparency, and justice, each comprising three items. Responses are rated on a 5-point Likert scale ranging from 1 (strongly disagree) to 5 (strongly agree), yielding a total score between 12 and 60. A higher score means greater ethical awareness of artificial intelligence. The scale demonstrates high internal consistency, with a Cronbach’s alpha of 0.88 (Ko and Leem 2021).

### 2.3 Data collection

The study's instruments were translated into Arabic using forward–backward translation by bilingual experts to ensure accuracy. A panel of seven specialists in medical-surgical and critical care nursing evaluated the Arabic version for clarity, cultural relevance, and content validity. A pilot test with 35 nurses (10% of the sample) confirmed the clarity and feasibility of the tools, with no revisions needed. For data collection, participants received a brief explanation and completed the questionnaires within 15–20 min.

### 2.4 Statistical analysis

Data were analyzed using both descriptive and inferential statistics. Sociodemographic characteristics of the participants were summarized using frequencies, percentages, means, and standard deviations. Descriptive statistics were also applied to examine the main study variables, including artificial intelligence acceptance, digital literacy, and artificial intelligence ethical awareness. Pearson correlation analysis was conducted to explore the relationships among these variables. To test the hypothesized structural relationships, Structural Equation Modeling (SEM) was performed using AMOS (Analysis of Moment Structures) with maximum likelihood estimation. AMOS is a statistical software package used for structural equation modeling (SEM), path analysis, and confirmatory factor analysis (CFA). It allows researchers to test complex relationships between observed and latent variables. Mediation effects were assessed using a bootstrapping procedure with 5000 resamples to estimate indirect effects and their 95% confidence intervals. Model fit was evaluated using several indices, including chi-square/degrees of freedom (*df*), Comparative Fit Index (CFI), Tucker–Lewis Index (TLI), Root Mean Square Error of Approximation (RMSEA), and Standardized Root Mean Square Residual (SRMR).

### 2.5 Ethical and institutional approvals

The study received ethical approval from the Research Ethics Committee (No FAN/161 on 24 February 2025). Written informed consent was secured from all participants using an informed voluntary form. All procedures conducted adhered to the ethical guidelines set by the relevant institutional and national committees overseeing human research, in alignment with the principles of the Declaration of World Medical Association (2013), as updated in 2000 (Serbaya et al., 2024).

## 3 Results

This section presents the findings of the study in four parts: (1) socio-demographic and professional characteristics of the participants; (2) descriptive statistics for digital literacy, artificial intelligence ethical awareness, and artificial intelligence acceptance; (3) correlation analysis among the main study variables; and (4) SEM assessing the direct and indirect effects of digital literacy on artificial intelligence acceptance, with artificial intelligence ethical awareness as a mediating variable.

### 3.1 Socio-demographic and professional characteristics of the participating nurses

Table 1: The study sample was predominantly composed of female and married nurses, with participants largely representing mid-career professionals. Most held a bachelor’s degree and had moderate clinical experience. Only a minority had received prior training in artificial intelligence. Participants were fairly distributed across medical, surgical, and critical care units, providing a diverse clinical representation.

**Table 1:** Sociodemographic characteristics and unit distribution of the study sample (*n* = 350).

Variable	Category	Frequency (*n*)	Percentage (%)
**Sex**	Male	150	42.9
	Female	200	57.1
**Age (years)**	18–29	85	24.3
	30–39	120	34.3
	40–49	90	25.7
	≥50	55	15.7
	**Mean ± SD**	37.6 ± 10.6	
**Marital Status**	Single	130	37.1
	Married	220	62.9
**Education Level**	Diploma	60	17.1
	Bachelor	265	75.7
	Postgraduate	25	7.1
**Clinical Experience**	6 months–5 years	100	28.6
	6–10 years	145	41.4
	>10 years	105	30.0
**Prior Artificial Intelligence Training**	Yes	95	27.1
	No	255	72.9
**Unit Type**	Medical Care	125	35.7
	Surgical Care	105	30.0
	Critical Care	120	34.3

**Note**: SD= standard deviation *n* = subsample size.

#### 3.1.1 Descriptive statistics for digital literacy, artificial intelligence ethical awareness, and artificial intelligence acceptance

Table 2 reflects that participants demonstrated a generally moderate level of artificial intelligence acceptance, with slightly greater confidence in the ease of use compared to its perceived usefulness, alongside a comparatively lower intention to adopt it in practice. Digital literacy levels were overall average, suggesting adequate but not advanced competencies across key digital domains. Additionally, participants exhibited a moderate level of ethical awareness regarding artificial intelligence, reflecting a balanced and cautious perspective toward issues such as accountability, safety, transparency, and justice in artificial intelligence utilization

**Table 2:** Descriptive statistics of artificial intelligence acceptance, digital literacy, and artificial intelligence ethical awareness among nurses.

Variable	Sub-domain	Mean ± SD
**Artificial Intelligence Acceptance**	Perceived Usefulness	17.8 ± 2.6
	Perceived Ease of Use	18.1 ± 2.7
	Intention to Use	11.2 ± 1.8
	**Overall**	**47.1 ± 5.2**
**Digital Literacy**	Media	35 ± 5.5
	Computer	37 ± 5.5
	Information	34 ± 5.5
	Visual	38 ± 5.5
	Communication	36 ± 5.5
	**Overall**	**180 ± 30**
**Artificial Intelligence Ethical Awareness**	Accountability	11.2 ± 1.6
	Safety	10.0 ± 1.8
	Transparency	10.0 ± 1.5
	Justice	10.5 ± 1.5
	**Overall**	**41.7 ± 5.6**

**Note**: SD= standard deviation.

#### 3.1.2 Correlation analysis among the main study variables

Table 3: The correlation analysis revealed significant positive relationships among the key study variables. Artificial intelligence acceptance was associated with both digital literacy and ethical awareness, while digital literacy also showed a positive relationship with ethical awareness. Overall, these findings suggest that higher acceptance of artificial intelligence is aligned with stronger digital competencies and greater ethical awareness, highlighting the interconnected nature of technological readiness and ethical considerations in nursing practice.

**Table 3:** Relationships between artificial intelligence acceptance, digital literacy, and artificial intelligence ethical awareness.

Variable	Mean ± SD	Total Artificial Intelligence Acceptance	Total Digital Literacy
**1. Total artificial intelligence Acceptance**	47.1 ± 5.2	—	
**2. Total Digital Literacy**	36.0 ± 5.5	0.42**	—
**3. Total Ethical Awareness**	42.5 ± 5.6	0.35**	0.46**

**Note**: SD= standard deviation.

#### 3.1.3 SEM assessing the direct and indirect effects of digital literacy on artificial intelligence acceptance, with artificial intelligence ethical awareness as a mediating variable

Table 4. The structural equation modelling results confirmed significant relationships among digital literacy, artificial intelligence ethical awareness, and artificial intelligence acceptance. Digital literacy emerged as a key predictor, influencing both ethical awareness and acceptance of artificial intelligence. In turn, ethical awareness significantly contributed to artificial intelligence acceptance, while also partially mediating the relationship between digital literacy and acceptance. Overall, we have highlighted that digital literacy played a central role in enhancing artificial intelligence adoption both directly and indirectly through strengthening nurses’ ethical awareness. The model demonstrated a good fit, supporting the robustness of the proposed relationships.

**Table 4:** Structural equation model of mediation results for digital literacy, artificial intelligence ethical awareness, and artificial intelligence acceptance.

Path	Standardized Estimate (β)	SE	CR	*p*-value	95% CI (Bootstrapped)
**Digital literacy → artificial intelligence ethical awareness**	0.46	0.05	9.20	<0.001	0.36 – 0.56
**Artificial intelligence ethical awareness → artificial intelligence acceptance**	0.28	0.06	4.67	<0.001	0.16 – 0.40
**Digital literacy → artificial intelligence acceptance (direct)**	0.29	0.06	4.83	<0.001	0.17 – 0.41
**Indirect effect: digital literacy → artificial intelligence ethical awareness → artificial intelligence acceptance**	0.13	0.03	4.33	<0.001	0.08 – 0.20
**Total effect: digital literacy → artificial intelligence acceptance**	0.42	0.06	7.00	<0.001	0.30 – 0.54
**Model fit indices:** χ²/df = 1.98, CFI = 0.95, TLI = 0.93, RMSEA = 0.057, SRMR = 0.045					

Note: β = standardized regression coefficient; SE = standard error; CR = critical ratio; CI = confidence interval; χ²/df = chi-square divided by degrees of freedom; CFI = comparative fit index; TLI = Tucker–Lewis index; RMSEA = root mean square error of approximation; SRMR = standardized root mean square residual; AI = artificial intelligence.

## 4 Discussion

We examined the relationship between digital literacy, nurses’ acceptance of artificial intelligence, and artificial intelligence-ethical awareness as a mediator. We have provided potentially-valuable insights into the factors influencing the adoption of artificial intelligence in nursing and highlighted the necessity of both ethical preparedness and digital competencies.

Nurses in this study demonstrated a moderate level of artificial intelligence acceptance, with perceived ease of use slightly higher than perceived usefulness and a relatively low intention to use artificial intelligence. These results are consistent with El Arab et al. (2025), who reported moderate acceptance of artificial intelligence among nurses, particularly when training and exposure were limited. Similarly, Serbaya et al. (2024) and Castagno and Khalifa (2020) found that healthcare professionals generally expressed positive attitudes and good awareness of artificial intelligence. In contrast, Elderiny and Ahmed (2024) found that nearly two-thirds of nurses demonstrated insufficient knowledge of artificial intelligence applications, reflecting ongoing challenges in preparedness. Likewise, Kaplan and Uçar (2024), Atalla et al. (2024), and Atalla et al., (2025) highlighted variability in attitudes, with a significant proportion of nurses reporting skepticism or concerns regarding artificial intelligence use.

We revealed a moderate positive correlation between artificial intelligence acceptance and digital literacy (*r* = 0.42, *p* < 0.01). This aligns with Asal et al. (2025), Rony et al. (2024), Rony et al. (2025), and Hoelscher et al. (2025), who highlighted digital competence as essential in the 21st century. Artificial intelligence literacy extends beyond technical proficiency to include ethical awareness and the ability to integrate artificial intelligence tools effectively into clinical workflows Chen et al., 2021;Danacı et al., 2025; Lifshits et al., 2024; Yang et al., 2024. Ethical awareness among participants was moderate, reflecting balanced but cautious views on accountability, safety, transparency, and justice. These results are consistent with Lifshits et al. (2024), Danacı et al. (2025), and Chen et al. (2021), who reported moderate ethical awareness, attitudes, and intention to use artificial intelligence among nursing students. This indicates that nurses more accepting of artificial intelligence were also more likely to develop ethical awareness, which enhances digital literacy, a relationship similarly observed in prior studies linking information competence to ethical sensitivity (Danacı et al., 2025; Chen et al., 2021).

Additionally, Sarıkahya et al. (2025) highlighted that faculty engagement with artificial intelligence tools influenced students’ readiness, emphasizing the importance of organizational and educational support for artificial intelligence acceptance. Boztepe et al. (2025) demonstrated that higher digital literacy was associated with more positive attitudes toward artificial intelligence among nursing students, corroborating our finding that digital competence facilitates acceptance. Özbay et al. (2026) showed that pediatric nurses’ ethical considerations directly affected artificial intelligence use, emphasizing that ethical awareness was a key mediator between competence and adoption. Together, these studies suggest that ethical preparedness is associated with digital literacy and artificial intelligence acceptance. Fostering artificial intelligence adoption in nursing may require a multifaceted approach: enhancing digital literacy, integrating ethics education, providing organizational support, and promoting trust through transparency and explainability. Interventions targeting knowledge, skills, and ethical competence may possibly be the most effective in building sustainable artificial intelligence adoption in nursing practice.

From the SEM, we observed that the findings strongly support the interconnected roles of digital literacy and ethical awareness in influencing participants’ acceptance of artificial intelligence in clinical practice. The significant direct path from digital literacy to artificial intelligence ethical awareness suggests that digitally competent nurses may be better equipped to recognize and evaluate ethical implications associated with artificial intelligence use. This result is consistent with Yang et al. (2024), who reported a moderate positive relationship (*r* = 0.30) between digital literacy and artificial intelligence ethics awareness among nursing students. Additionally, digital literacy was significantly associated with artificial intelligence acceptance, suggesting that digital proficiency not only enhances ethical understanding but also may build confidence in using artificial intelligence tools effectively.

The mediating role of ethical awareness adds nuance to this relationship. The indirect effect indicated that ethical awareness partially mediated the link between digital literacy and artificial intelligence acceptance. Digitally-literate nurses tend to be more ethically aware, which fosters openness toward artificial intelligence integration (Yang et al., 2024). This underscores the importance of ethical preparedness in potentially shaping positive attitudes toward the adoption of artificial intelligence. However, the presence of partial mediation indicates that additional factors, such as trust, perceived usefulness, institutional support, and user anxiety, may also influence this relationship (Yang et al. 2024).

Ultimately, we have demonstrated uniquely that artificial intelligence ethical awareness mediated the relationship between digital literacy and artificial intelligence acceptance within a sample of clinical nurses, highlighting that technical competence alone may be insufficient for effective adoption. The findings underscore that nurses' ethical preparedness may actively bridge the gap between digital skills and practical artificial intelligence use, with moderate ethical awareness and artificial intelligence acceptance observed in this sample.

These results may have implications for nursing education, emphasizing consideration of the integration of ethical reasoning, accountability, transparency, and fairness into curricula, as supported by recent studies in nursing artificial intelligence (Sarıkahya et al., 2025; Boztepe et al. (2025); Özbay et al., 2026). At the organizational level, fostering artificial intelligence adoption requires not only technical training but also ethical governance, transparent policies, and institutional support. By extending technology acceptance models to include ethical awareness as a critical mediating factor, we intended to provide a practical framework for both educational and institutional strategies, highlighting that digital literacy and ethical competence are associated with responsible, effective, and patient-centered artificial intelligence integration in nursing practice (Reddy et al., 2023; Ko and Leem, 2021& World Medical Association, 2013).

### 4.1 Limitations

Conducting the study in a single healthcare setting limits the generalizability of the findings to other healthcare institutions, regions, and countries with different organizational cultures, healthcare infrastructures, technological readiness, and nursing workforce characteristics. Additionally, the cross-sectional design precludes the establishment of causal or directional relationships among the studied variables and captures data at a single point in time. The use of convenience sampling may have introduced selection bias, with digitally inclined nurses more likely to participate. Furthermore, self-reported data may be affected by social desirability or recall bias. Therefore, caution should be exercised when interpreting and applying the findings to international contexts, particularly in countries with differing levels of artificial intelligence integration, digital health maturity, and cultural perspectives toward technology use in healthcare. Future researchers should employ multi-site and cross-national designs, probability sampling techniques, and objective assessment measures to enhance the validity and generalizability of the findings across diverse healthcare systems and cultural settings.

### 4.2 Implication for practice

Prioritizing nurses’ digital literacy may be important, as it was associated with both ethical awareness and acceptance of artificial intelligence technologies. To support responsible artificial intelligence adoption, ongoing professional development should consider combining digital competency with artificial intelligence ethics training, possibly enabling Egyptian nurses to make informed and accountable decisions in clinical care, patient monitoring, and care planning concepts collectively referred to as artificial intelligence-supported care.

Nurse leaders and educators may be able to reinforce these competencies through simulation and case-based learning, digital resources, and policies that promote the ethical use of artificial intelligence. Strengthening nurses’ digital literacy and ethical awareness may ensure that artificial intelligence is applied in ways that protect patient safety, privacy, and fairness, recognize potential biases, and maintain professional accountability, thereby supporting safer, more effective, and ethically responsible integration of artificial intelligence in clinical practice and enhancing overall acceptance of artificial intelligence technologies among nurses.

## 5 Conclusion

Digital literacy and artificial intelligence ethical awareness may be key factors influencing nurses’ acceptance of artificial intelligence in clinical practice. Higher digital literacy may enhance both ethical awareness and willingness to adopt artificial intelligence, with ethical understanding partially mediating this relationship. We suggest the need for training programs that integrate digital skills and artificial intelligence ethics to prepare nurses for safe and responsible artificial intelligence use in healthcare.

## Credit contribution statement

Wafaa Awad and Eshrak Hashem: Writing – review & editing, Validation, Project administration, Methodology, Investigation, Data curation, Conceptualization. Nadia Awad, Heba Ashour, and Engy AbdlRhman: Writing – review & editing, Validation, Project administration, Methodology, Data curation, Conceptualization. Mai Yassen: Formal analysis and Methodology. Hadaiea Ismail: Formal analysis and writing, review and editing. Narges Syam: formal analysis, validation, writing review, and editing. Funding Statement The author(s) received no financial support for the research, authorship, and/or publication of this article. Conflict of Interest Disclosure The authors declare no conflicts of interest. Ethical Considerations This study was approved by the Research Ethics Committee, Modern University for Technology and Information (Approval No FAN/161 on 24 February 2025). Consent Statement Informed consent was obtained from all participants, with assurances of confidentiality and privacy. Data Availability Statement The datasets generated and analyzed during the current study are not publicly available due to restrictions on the participants’ privacy and are available from the corresponding author on reasonable requests. Acknowledgements The authors acknowledge all nurses who exhibited great patience in completing the questionnaires, to whom we express our deepest gratitude for their help and support. Authorship change request Important information – please read before continuing. How to use this form • This form is to be completed by the Corresponding Author to request any change in authorship (additions, removals, or reordering) after the submission of a manuscript, including changes in Corresponding Authors, if any. • Changes to the author list must not be made in the journal’s Editorial Manager system without submitting this form. Unauthorized authorship changes will result in the rejection of your submission, or retraction if the article has already been published. • Authorship changes are not allowed after manuscript acceptance. It is also not possible to submit this form after your manuscript has been rejected, for any reason. • Do not use this form for name changes or corrections. • This form consists of three parts, all of which should be completed prior to submission. A note about author disputes The publisher and editor cannot investigate or mediate any authorship disputes. If you are unable to obtain agreement from all authors, including those you intend to remove, we recommend seeking guidance from your institution. We will not consider your change request and will not proceed with the publication of your manuscript until all outstanding authorship disputes are resolved. Before completing the form • All authors should carefully review the “Duties of Authors” section of the Elsevier publishing ethics policy, particularly the sections on: o Authorship of the paper o The use of generative AI and AI-assisted technologies in scientific writing and in figures, images and artwork • Please also carefully review the submission journal’s “Guide for authors” (this might also be referred to as “Instructions for authors”), because some journals may have additional authorship criteria (an example is the ICMJE guidelines for authorship). • Be prepared to enter the full name, email address, and institution for every author on the manuscript. This information must match the author list in the submission system. How to submit this form After completing this form according to the above instructions, it should be submitted through the journal’s Editorial Manager system with your revised manuscript via the “Submissions Needing Revision” link. Advancing human progress together 1 (continued from the previous page) In the “Attach Files” step, use the “Cover letter” item type and in the description, type “Authorship change request form” and upload this form. Make corresponding changes in the Manuscript Data step before completing your revision submission. For additional guidance, view the tutorial on using Editorial Manager for this process in the Journal Publishing Support Center. Once submitted, the information in this form will become part of the revision submission record. Part 1. General submission information To be completed by the Corresponding Author. Manuscript details Journal title International Journal of Nursing Studies Advances Manuscript and/ or article number IJNSA-D-26-00103R1 Manuscript title The Role of Digital Literacy and Ethical Awareness in Nurses’ Artificial Intelligence Acceptance: A Cross-sectional Questionnaire Survey Change(s) requested (check all that apply) Add new author(s) Remove author(s) ✔ Change the Corresponding Author Change the order of authors* * If ONLY changing order of existing authors, go directly to Part 3. Do not complete Part 2. Advancing human progress together 2 Author’s Individual contributions (required for author additions only) see CRediT Contributor Roles Taxonomy Part 2. Indicate author(s) to be added or removed For each author to be added or removed complete one template below.** In the “Reason for this change” section, please include as much detail as possible so we can evaluate if the change is approved. At a minimum, this should include explanations for both: • why the change is being requested, and • why the author was/was not included in the original author list. Important Note: If this part is either not provided, incomplete, or the reasons provide insufficient detail or do not address the points above, your request will be denied, and your submission may be rejected. Given/first name(s) Family/last name Email address Dr. Nadia Hassan Ali Awad Nadia.hassan@alexu.edu.eg Institution: Nursing Administration Department, Faculty of Nursing, Alexandria University, Alexandria, Egypt; Nursing program, Batterjee Medical College, Jeddah 21,442, Saudi Arabia Change(s) requested for Remove author Add new author Make Corresponding Author this author Conceptualization Data curation Formal analysis Funding acquisition Investigation Methodology Project administration Resources Software Supervision Validation Visualization Writing – original draft Writing – review & editing Reason for this change The reason for requesting the change of the corresponding author is to facilitate communication and handling of the revision and publication process. By mutual agreement among all co-authors, New Corresponding Author Dr. Nadia Hassan Ali Awad will assume responsibility for all future correspondence related to the manuscript. All authors have agreed to this change. Thank you for your understanding and consideration. 2.1 Author change information Given/first name(s) Family/last name Email address Institution Change(s) requested for this author Remove author Add new author Make Corresponding Author Conceptualization Data curation Formal analysis Funding acquisition Investigation Methodology Project administration Resources Software Supervision Validation Visualization Writing – original draft Writing – review & editing Given/first name(s) Family/last name Email address Institution Change(s) requested for this author Remove author Add new author Make Corresponding Author Conceptualization Data curation Formal analysis Funding acquisition Investigation Methodology Project administration Resources Software Supervision Validation Visualization Writing – original draft Writing – review & editing Advancing human progress together 4 2.2 Author change information Author’s Individual contributions (required for author additions only) see CRediT Contributor Roles Taxonomy Reason for this change 2.3 Author change information Author’s Individual contributions (required for author additions only) see CRediT Contributor Roles Taxonomy Reason for this change 2.5 Author change information Funding acquisition Investigation Methodology Project administration Resources Software Supervision Validation Visualization Writing – original draft Writing – review & editing Reason for this change Given/first name(s) Family/last name Email address @ Institution Change(s) requested for this author Remove author Add new author Make Corresponding Author’s Individual contributions (required for author additions only) see CRediT Contributor Roles Taxonomy Conceptualization Data curation Formal analysis Funding acquisition Investigation Methodology Project administration Resources Software Supervision Validation Visualization Writing – original draft Writing – review & editing Reason for this change Given/first name(s) Family/last name Email address @ Institution Change(s) requested for this author Remove author Add new author Make Corresponding Author ** If you have >5 author changes, add additional pages of the above template to provide their details. Advancing human progress together 5 Conceptualization Data curation Formal analysis Author’s Individual contributions (required for author additions only) see CRediT Contributor Roles Taxonomy 2.4 Author change information Agreement of removed authors Part 3. Confirm author order and agreement 1. Listing removed and proposed authors Please enter the full name and details for all the removed authors in the table below, “Agreement of removed authors.” In the next table, “Proposed author list,” enter the full name and details of all the proposed authors in the order that they should appear in the publication. “Full name” is the author’s name as it appears in the submission system author list. 2. Gathering signatures of all authors This form must be signed individually by every author, including both added and removed authors. The only exception is for cases where consortia group authorship was declared at submission, in which case the corresponding author may sign on behalf of the group. While handwritten signatures are acceptable, we highly encourage the use of electronic signature software (DocuSign, Adobe Sign, Dropbox Sign, or similar) with valid e-signatures. These digital signatures should reflect your institutional information and email as provided in the author list below. By signing this form all authors agree: 1) that they have read and acknowledge the publishing ethics policies linked in the “Important Information” section of this form, and; 2) to the addition and/or removal of the authors listed in Section 2 and to the revised order of the author list in this Section 3, and; 3) that all information provided accurately reflects the authorship of the article. Full name Email address Signature Date Wafaa Hassan Ali Awad wafaa.hassan@alexu.edu.eg Nadia Hassan Ali Awad nadia.hassan@alexu.edu.eg Heba Mohammed Alanwer As haba.alanwer@alexu.edu.eg Engy AbdlRhman Khamis engy.abdelrhman@nur.mti.edu. Mai Mohammed Yaseen myasin@kau.edu.sa May 14, 2026 May 14, 2026 May 14, 2026 May 14, 2026 May 14, 2026 Add additional page(s) if needed. Advancing human progress together 6 Part 3. Confirm author order and agreement 1. Listing removed and proposed authors Please enter the full name and details for all the removed authors in the table below, “Agreement of removed authors.” In the next table, “Proposed author list,” enter the full name and details of all the proposed authors in the order that they should appear in the publication. “Full name” is the author’s name as it appears in the submission system author list. 2. Gathering signatures of all authors This form must be signed individually by every author, including both added and removed authors. The only exception is for cases where consortia group authorship was declared at submission, in which case the corresponding author may sign on behalf of the group. While handwritten signatures are acceptable, we highly encourage the use of electronic signature software (DocuSign, Adobe Sign, Dropbox Sign, or similar) with valid e-signatures. These digital signatures should reflect your institutional information and email as provided in the author list below. By signing this form all authors agree: 1) that they have read and acknowledge the publishing ethics policies linked in the “Important Information” section of this form, and; 2) to the addition and/or removal of the authors listed in Section 2 and to the revised order of the author list in this Section 3, and; 3) that all information provided accurately reflects the authorship of the article. Agreement of removed authors Full name Email address Signature Date Hadaiea Ismail Abo Baker Ismail Hadaieaismail@alexu.edu.eg 14 May 2026 Narges Mohammed Mohammed Syam narges-seyam@alexu.edu.eg 14 May 2026 Eshrak Salama Hashem eshrak.hashem@alexu.edu.eg May 14, 2026 Add additional page(s) if needed. Advancing human progress together 6 Proposed author list Order Full name 01 Wafaa Hassan Ali Awad Email address wafaa.hassan@alexu.edu.e Signature Date 14-5-2026 02 Nadia Hassan Ali Awad nadia.hassan@alexu.edu.e 14-5-2026 03 Heba Mohammed Alanwer Ash haba.alanwer@alexu.edu.e 14-5-2026 04 Engy AbdlRhman Khamis ngy.abdelrhman@nur.mti.e 14-5-2026 05 Mai Mohammed Yaseen myasin@kau.edu.sa 14-5-2026 06 Hadaiea Ismail Abo baker Hadaieaismail@nec.edu.sa 14-5-2026 07 Narges Mohammed Mohamme narges-seyam@alexu.edu.e 14-5-2026 08 Eshrak salama Hashem eshrak.hashem@alexu.edu 14-5-2026 09 @ 10 @ 11 @ 12 @ 13 @ 14 @ 15 @ 16 @ 17 @ 18 @ 19 @ 20 @ 21 @ 22 @ 23 @ 24 @ 25 @ Add additional page(s) if needed. Advancing human progress together 7

## CRediT authorship contribution statement

**Wafaa Hassan Ali Awad:** Methodology. **Nadia Hassan Ali Awad:** Formal analysis, Data curation. **Heba Mohammed Alanwer Ashour:** Supervision. **Engy AbdlRhman Khamis:** Conceptualization. **Mai Mohammed Yassen:** Methodology, Formal analysis. **Hadaiea Ismail Abo baker Ismail:** Writing – review & editing, Formal analysis. **Narges Mohammed Mohammed syam:** Writing – review & editing, Validation, Formal analysis. **Eshrak salama Hashem:** Writing – review & editing, Writing – original draft.

## Declaration of competing interest

The authors declare no conflicts of interest.
